# Association between neutrophil-to-lymphocyte ratio and diabetic retinopathy in patients with type 2 diabetes: a cohort study

**DOI:** 10.3389/fendo.2024.1396161

**Published:** 2024-07-11

**Authors:** Yuchen Tang, Li Li, Jialin Li

**Affiliations:** Department of Endocrinology and Metabolism, The First Affiliated Hospital of Ningbo University, Ningbo, China

**Keywords:** neutrophil-to-lymphocyte ratio, diabetic retinopathy, type 2 diabetes, inflammation, cohort study

## Abstract

**Background:**

Chronic inflammation is implicated in the development of diabetic retinopathy (DR). The neutrophil-to-lymphocyte ratio (NLR) is a marker of systemic inflammation that has been linked to cardiovascular and diabetic kidney diseases. However, the link between NLR and DR remains unclear. As such, this study investigated the association between NLR and DR in Chinese patients.

**Method:**

A total of 857 adults diagnosed with type 2 diabetes mellitus (T2DM) without DR at baseline between 2018 and 2021, from a single center in Ningbo, China, were included. Baseline clinical data, including age, sex, T2DM duration, hypertension, smoking, drinking, glycated hemoglobin level, lipid profile, renal function, and NLR, were recorded and analyzed. Cox proportional hazard regression analysis was used to assess the association between NLR and the risk for incident DR.

**Results:**

During a median follow-up of 3.0 years, 140 patients developed DR. The multivariable-adjusted hazard ratio (HR) for incident DR across ascending NLR quartiles (≤1.46 [reference], 1.47–1.90, 1.91–2.45 and > 2.45) were 1.000, 1.327 (95% confidence interval [CI] 0.754–2.334), 1.555 (95% CI 0.913–2.648) and 2.217 (95% CI 1.348–3.649), respectively. For each 1-standard deviation increase in NLR, the risk for DR increased by 29.2% (HR 1.292 [95% CI 1.112–1.501) after adjusting for confounding factors.

**Conclusion:**

Results revealed that a higher NLR at baseline was associated with an increased risk for incident DR. NLR has the potential to be an inexpensive, reliable, and valuable clinical measure that merits further exploration in future studies.

## 1 Introduction

Diabetes mellitus (DM) is a slowly progressive metabolic illness characterized by long-standing hyperglycemia that can cause various complications (1). Diabetic retinopathy (DR) is one of the most prevalent complications and a leading cause of blindness among working-age adults, seriously affecting patient quality of life and increased financial burden (2, 3). The early detection and prevention of DR are crucial to avoid blindness.

There is accumulating evidence that chronic low-grade inflammation has a particular influence on the development of DM and metabolic syndrome (4). Chronic inflammation is a hallmark of type 2 DM (T2DM), possibly due to insulin resistance and impaired glucose metabolism (5).

DR is a progressive microvascular and neurodegenerative disease that occurs gradually with years of poor glycemic control (6). Although clinical signs, such as acellular capillaries, retinal nonperfusion, and ischemia appear later, early changes involve persistent low-grade leukocyte activation triggered by metabolic dysregulation (7). This activation disrupts the retinal neurovascular unit and its cellular components (7).

The neutrophil-to-lymphocyte ratio (NLR), which is the synthesis of two distinct but complementary immune pathways of the innate and adaptive cellular immune responses, has been studied extensively as an inflammatory marker in many malignant and benign diseases (8). Western studies have reported that an elevated NLR is independently linked to increased cardiovascular death and all-cause mortality in patients with diabetes, as well as a higher risk for diabetic kidney disease (9, 10). However, the relationship between the NLR and DR remains unclear, with only a limited number of cross-sectional studies exploring this relationship (11, 12). As such, the present cohort study aimed to evaluate whether the presence of DR is correlated with NLR in Chinese patients.

## 2 Methods

### 2.1 Study population

In this retrospective cohort study, patients diagnosed with T2DM, who attended the Department of Endocrinology and Metabolism, First Affiliated Hospital of Ningbo University, and participated in the multihospital-based program of the National Metabolic Management Center (MMC) were recruited from March 2018 to December 2021. The MMC program, led by Ruijin Hospital in Shanghai (China), followed a standard protocol for the professional management and follow-up of patients with DM (13).

The inclusion criteria were as follows: age 18–75 years; confirmed T2DM diagnosis (1999 World Health Organization criteria (14)); absence of DR at baseline; and available annual fundus photography data. Exclusion criteria were as follows: history of other fundus diseases; history of hematological diseases; acute diabetic complications, such as diabetic ketoacidosis; other concomitant conditions influencing glucose metabolism (acute infection, chemotherapy, use of corticosteroids); history of cancer, heart failure, end-stage renal disease, chronic liver disease; or loss to follow-up or short follow-up (<2 years). In total, data from 857 patients were included in the final analysis. This study adhered to the Declaration of Helsinki and was approved by the Ethics Committees of both hospitals (Ruijin Hospital: 2017 No. 42; Ningbo University: 2019-R057). Informed consent was obtained from all participants.

### 2.2 Baseline clinical and biological characteristics

Demographic information (age, sex, and T2DM duration), lifestyle habits (history of smoking and alcohol consumption), and medical histories were obtained using standardized MMC questionnaires. Trained nurses measured the height, weight, and waist circumference of the participants. Body mass index was calculated as the ratio of weight (kg) to height squared (m^2^). Visceral fat area was assessed using dual bioelectrical impedance analysis (DUALSCAN HDS-2000, Omron, Japan).

Hypertension was defined as systolic blood pressure (SBP) ≥140 mmHg and/or diastolic blood pressure (DBP) ≥90 mmHg or antihypertensive medication use (15). Dyslipidemia was defined as fasting triglycerides (TG) ≥1.7 mmol/L, high-density lipoprotein cholesterol (HDL-c) ≤1.04 mmol/L (males) or ≤1.30 mmol/L (females), and low-density lipoprotein cholesterol (LDL-c) ≥ 2.60 mmol/L determined by enzymatic assays (AU5800, Beckman Coulter, USA) or on lipid-lowering agents (16).

After an overnight fast, venous blood and urine samples were collected in the morning. Complete blood count with differential analysis was performed using an automated analyzer (Sysmex XN-9000, Kobe, Japan). The NLR was calculated. Glycated hemoglobin (HbA1c), fasting plasma glucose, and fasting insulin levels were measured using established methods (D-10 Hemoglobin Analyzer, Bio-Rad, Hercules, CA, USA; glucose oxidase method; chemiluminescence immunoassay). The Homeostatic Model Assessment for Insulin Resistance (i.e., “HOMA-IR”) index, reflecting insulin resistance, was calculated using a standard equation: fasting blood glucose (FBG, mmol/L) × fasting insulin (mU/L) ÷ 22.5 (17). Serum creatinine and uric acid levels were measured using standard enzymatic methods (AU5800, Beckman Coulter, Brea, CA, USA). The estimated glomerular filtration rate (eGFR) was determined using the Chronic Kidney Disease Epidemiology Collaboration (CKD-EPI) algorithm (18). The urinary albumin-to-creatinine ratio (UACR) was calculated from the ratio of urinary albumin to creatinine measured in spot urine samples using immunonephelometry (albumin) and enzymatic methods (creatinine).

### 2.3 Outcome

The primary outcome was incident DR. All patients underwent non-mydriatic fundus photography at the first visit and were re-evaluated for DR during each annual visit (Topcon, Tokyo, Japan) (19). Two images were captured per eye, centered on the macula and optic nerve at 45°, following established protocols for effective DR screening (20). DR was diagnosed based on the International Classification of DR (21).

### 2.4 Statistical analysis

Normally distributed continuous variables are expressed as mean ± standard deviation (SD) and were compared using independent-samples *t*-tests. Continuous variables with non-normal distribution are expressed as median and interquartile range (i.e., 25th percentile, 75th percentile) and compared using the Mann–Whitney U test. Categorical variables are expressed as frequency (percentage) and compared using the chi-squared test to assess differences in baseline characteristics between patients with incident DR and those without DR (NDR) in the T2DM group.

Cox proportional hazards regression was applied to evaluate the association between NLR, as either categorical (quartile 1, reference group: ≤1.46; quartile 2, 1.47–1.90; quartile 3, 1.91–2.45; and quartile 4, > 2.45) or continuous variable (per 1-standard deviation [SD] change), and the occurrence of DR (yes versus [vs.] no). The analyses were first performed without adjustment (model 1) and were then adjusted for age, sex (model 2), T2DM course, smoking history, drinking history, BMI, SBP, TG, HbA1c, FBG, fasting insulin, uric acid, eGFR, and UACR (model 3). Covariates for the multivariate model were chosen based on the literature and univariate analysis. Differences with a two-tailed *P*<0.05 were considered to be statistically significant. Data analysis was performed using SPSS version 27.0 (IBM Corp., Armonk, NY, USA) for Windows (Microsoft Corp., Redmond, WA, USA).

## 3 Results

A total of 857 (560 male, 297 female) subjects were included in the final analysis. Over a median 3-year follow-up period, 140 patients developed DR. Baseline characteristics stratified according to incident DR are summarized in **Table 1**. The median NLR was 2.21 in patients who developed the retinopathy outcome (i.e., DR), compared with 1.86 in the patients not developing retinopathy (i.e., NDR) (*P*<0.001). The 25th, 50th, and 75th NLR percentiles were 1.46, 1.90, and 2.45, respectively. Briefly, subjects with incident DR had a notably higher mean age (52.4 vs. 47.3 years; *P*<0.001), a longer diabetes course (median 89.5 vs. 42 months; *P*<0.001), higher levels of HbA1c (median 7.75% vs. 7.3%; *P*=0.001), FBG (median 8.60 vs. 7.63 mmol/L; *P*=0.046), NLR and lower levels of fasting insulin (median 8.26 vs. 9.85 mU/L; *P*=0.015), TGs (median 1.27 vs. 1.49 mmol/L; *P*=0.048), uric acid (mean 316.10 vs. 336.51μmol/L; *P*=0.010), and eGFR (mean 104.79 vs. 107.72 mL/min/1. 73m^2^; *P*=0.043) than those without incident DR.

**Table 1:** Clinical characteristics of patients grouped by the incident diabetic retinopathy.

	Non-DR (n = 717)	DR (n =140)	*P* value
Age, years	47.3 ± 11.2	52.4 ± 10.9	<0.001
Male, n (%)	470 (65.6)	90 (64.3)	0.774
Diabetes course, months	42 (6, 97)	89.5 (24, 146)	<0.001
Smoking history, n (%)	317 (44.2)	58 (41.4)	0.544
Drinking history, n (%)	416 (58.0)	69 (49.3)	0.057
SBP, mmHg	131.75 ± 17.39	134.75 ± 18.89	0.066
DBP, mmHg	80.63 ± 11.00	79.37 ± 11.23	0.216
Hypertension, n (%)	387 (54.0)	79 (56.4)	0.594
Dyslipidemia, n (%)	639 (89.1)	117 (83.6)	0.062
BMI, kg/m^2^	25.60 ± 3.93	25.09 ± 3.01	0.081
Waist Circumference, cm	89.59 ± 10.86	88.50 ± 9.74	0.269
VFA, cm^2^	95.78 ± 41.42	92.01 ± 40.53	0.334
HbA1c, %	7.3 (6.3, 8.9)	7.75 (6.8, 9.75)	0.001
Fasting blood glucose, mmol/L	7.63 (6.52, 9.61)	8.60 (6.61, 10.37)	0.046
Fasting insulin, mU/L	9.85 (6.51, 14.93)	8.26 (4.91, 13.37)	0.015
HOMA-IR	3.52 (2.19, 5.80)	3.31 (1.91, 5.57)	0.161
Triglycerides, mmol/L	1.49 (0.99, 2.21)	1.27 (0.94, 2.10)	0.048
Total cholesterol, mmol/L	4.70 ± 1.18	4.56 ± 1.14	0.197
HDL-C, mmol/L	1.16 ± 0.27	1.20 ± 0.29	0.133
LDL-C, mmol/L	3.07 ± 0.88	2.99 ± 0.89	0.346
Uric acid, μmol/L	336.51 ± 85.53	316.10 ± 83.05	0.010
Creatinine, μmol/L	63.70 ± 15.65	62.33 ± 17.71	0.357
eGFR, mL/min/1. 73m^2^	107.72 ± 15.19	104.79 ± 17.70	0.043
UACR, mg/g	10.63 (5.66, 26.53)	12.00 (7.03, 26.95)	0.093
NLR	1.86 (1.43, 2.39)	2.21 (1.59, 2.71)	<0.001

DR, diabetic retinopathy; SBP, systolic blood pressure; DBP, diastolic blood pressure; BMI, Body mass index; VFA, visceral abdominal fat area; HbA1c, glycated hemoglobin; HOMA-IR, insulin resistance index; HDL-C, high-density lipoprotein cholesterol; LDL- C, low-density lipoprotein cholesterol; Cr, blood creatinine; eGFR, glomerular filtration rate; UACR, urinary albumin-to-creatinine ratio; NLR, neutrophil-to-lymphocyte ratio.

The multivariable-adjusted (age, sex, diabetes course, smoking history, drinking history, BMI, SBP, TG, HbA1c, FBG, fasting insulin, uric acid, eGFR, and UACR) hazard ratios (HRs) for incident DR across ascending NLR quartiles (≤1.46 [reference], 1.47–1.90, 1.91–2.45 and > 2.45) were 1.000, 1.327 (95% confidence interval [CI] 0.754–2.334), 1.555 (95% CI 0.913–2.648) and 2.217 (95% CI 1.348–3.649), respectively (*P*=0.011) (**Table 2**). For each 1-SD increase in NLR, the risk for DR increased by 29.2% (HR 1.292 [95% CI 1.112–1.501]; *P*=0.001) after adjusting for confounders (**Table 3**). Age, diabetes course, and HbA1c level were also independently associated with incident DR (**Table 3**).

**Table 2:** Hazard ratios for diabetic retinopathy according to neutrophil-to-lymphocyte ratio as categorical variable.

	NLR quartile				
	Q1 (≤1.46)	Q2 (1.47–1.90)	Q3 (1.91–2.45)	Q4 (> 2.45)	*P* value
No. of participants	215	214	215	213	–
No. of cases	24	26	38	52	–
HRs for incident DR (95% CI)					
Model 1	1.000	1.156 (0.664–2.013)	1.621 (0.972–2.702)	2.507 (1.545–4.068)	<0.001
Model 2	1.000	1.163 (0.668–2.026)	1.640 (0.983–2.734)	2.293 (1.411–3.726)	0.002
Model 3	1.000	1.327 (0.754–2.334)	1.555 (0.913–2.648)	2.217 (1.348–3.649)	0.011

Model 1: unadjusted; Model 2: adjusted for age and sex; Model 3: adjusted for age, sex, diabetes course, smoking history, drinking history, body mass index, systolic blood pressure, triglycerides, glycated hemoglobin, fasting blood glucose, fasting insulin, uric acid, glomerular filtration rate and urinary albumin-to-creatinine ratio. DR, diabetic retinopathy; NLR, neutrophil-to-lymphocyte ratio; HR, hazard ratio; CI, confidence interval.

**Table 3:** The adjusted multivariable cox regression for diabetic retinopathy according to neutrophil-to-lymphocyte ratio as continuous variable.

	HR	95% CI	*P* value
Age	1.034	1.015, 1.052	<0.001
Diabetes course	1.005	1.002, 1.007	<0.001
HbA1c	1.198	1.107, 1.298	<0.001
NLR	1.292	1.112, 1.501	0.001

NLR, neutrophil-to-lymphocyte ratio; HR, hazard ratio; CI, confidence interval.

## 4 Discussion

The present cohort study identified a positive correlation between NLR, a composite metric for chronic inflammation, and the incidence of DR in patients with T2DM. Each 1-SD increase in the NLR corresponded to a 29% increased risk for developing DR.

Chronic low-grade inflammation and endothelial dysfunction are well-established contributors to insulin resistance, T2DM, and other microvascular and macrovascular complications (12). Inflammatory responses triggered by hyperglycemia play a key role in vascular dysfunction. Hyperglycemia induces the production of reactive oxygen species and upregulates the expression of pro-inflammatory and pro-coagulant factors, which promote adhesion between white blood cells and endothelial cells (22). The accumulation of leukocytes in capillaries disrupts the normal structure between the retinal endothelial cells and pericytes, leading to a damaged blood-retinal barrier. Excessive accumulation of leukocytes can cause vascular blockage and leakage, which in turn exacerbate damage to the blood-retinal barrier (23). Overall, the emergence of inflammatory mediators and disturbances in the balance between angiogenesis-related factors promote the development of DR (24). This results in increased retinal vascular permeability, neurodegeneration, damage to the blood-reticulum barrier, and neovascularization, ultimately leading to diabetic macular edema and proliferative DR (24). NLR reflects both cellular immune activation and systemic inflammatory response, acting as an indicator of the balance between two key immune system components: neutrophils, active non-specific inflammatory cells, lymphocytes, and protective or regulatory cells (8). The NLR has emerged as a promising marker in this context.

Our results are consistent with those of most previous studies that have explored the relationship between the inflammatory index and diabetic complications. This aligns with a case-control study from Turkey (25), where NLR levels were higher in patients with diabetes and DR than in those without DR. While some studies, such as those from northern China (26), reported higher NLR in patients with DR, and the investigators did not find NLR to be an independent risk factor. Moreover, another cross-sectional study from Turkey that analyzed 114 patients with T2DM reported that NLR was not independently associated with DR (27). Potential explanations for these variations include the larger sample size, differences in subject characteristics, and variations in lifestyle factors.

Several studies have supported the link between NLR and diabetic microvascular complications. A cross-sectional study observed a positive association between a higher NLR and the occurrence of both nephropathy and retinopathy in an Indian population (12). Additionally, an elevated NLR was correlated with diabetic nephropathy in other studies (10, 28) and even served as an independent predictor of kidney function decline in individuals with DM (29). DR and nephropathy share common underlying mechanisms and often co-occur (30). Given this shared etiology and the association between NLR and both complications, NLR may emerge as a valuable predictor and prognostic marker for both DR and nephropathy.

The present study had several limitations, the first of which was its retrospective cohort design, which precluded the determination of a causal relationship between the biomarkers and outcomes. Second, we did not analyze correlations stratified according to DR severity (i.e., non-proliferative and proliferative DR), because only 1 participant developed proliferative DR. Third, lifestyle, socioeconomic, and pharmacological interventions were not considered in this study. Additionally, the relatively short follow-up period limited our ability to fully assess the long-term impact of NLR on DR progression. Future studies with larger cohorts and extended follow-up periods are essential to validate our findings and explore these associations more thoroughly.

In summary, results of the present study indicate that the incidence of DR is associated with a higher NLR. These findings may provide instructive options for clinical practice. According to statistics, there are an estimated 141 million patients with diabetes in China, and the number of patients with DR has exceeded 19 million (31, 32). With an aging population and a high incidence of DM, dealing with visual impairment and blindness is a huge challenge. Screening for DR offers a preferable solution to decrease preventable blindness and the medical economic burden. According to these guidelines, routine screening every to 1–2 years is recommended. Based on the results of our study, we recommend that patients with diabetes and higher NLR should undergo a shorter period of regular screening for DR (e.g., 6 months). Eye examination by fundus photography using a non-mydriatic fundus camera costs < $2 in our hospital, and early detection of DR could save thousands from medical expenditures for the treatment of DR.

## 5 Conclusion

In conclusion, results of the present study indicated that, among patients with T2DM, a higher NLR was associated with an increased risk for incident DR. Given the advantages of NLR in terms of stability and accessibility, this result further supports that NLR could be a valuable clinical measure. Future studies with various populations and designs are necessary to further explore the potential value of the NLR in predicting diabetes-related outcomes.

## Data availability statement

The raw data supporting the conclusions of this article will be made available by the authors, without undue reservation.

## Ethics statement

The studies involving humans were approved by Research Ethics Committee of Ruijin Hospital (2017 No. 42) and the First Affiliated Hospital of Ningbo University (2019-R057). The studies were conducted in accordance with the local legislation and institutional requirements. The participants provided their written informed consent to participate in this study.

## Author contributions

YT: Conceptualization, Data curation, Funding acquisition, Investigation, Methodology, Software, Supervision, Writing – original draft, Writing – review & editing. LL: Funding acquisition, Writing – review & editing. JL: Funding acquisition, Writing – review & editing.
